# Influence of Biogenic Material Content on the Biodegradability of Styrene-Butadiene Composites with Incorporated *Chlorella vulgaris* Biomass

**DOI:** 10.3390/polym16091241

**Published:** 2024-04-29

**Authors:** Marius Bumbac, Cristina Mihaela Nicolescu, Traian Zaharescu, Costel Bumbac, Elena Elisabeta Manea, Ioana Alexandra Ionescu, Ion Valentin Gurgu, Bogdan-Catalin Serban, Octavian Buiu, Crinela Dumitrescu

**Affiliations:** 1 Faculty of Science and Arts, Valahia University of Targoviste, 13 Aleea Sinaia, 130004 Targoviste, Dambovita, Romania; marius.bumbac@valahia.ro (M.B.); crinela.dumitrescu@valahia.ro (C.D.); 2 Institute of Multidisciplinary Research for Science and Technology, Valahia University of Targoviste, 13 Aleea Sinaia, 130004 Targoviste, Dambovita, Romania; traian_zaharescu@yahoo.com (T.Z.); valentin.gurgu@valahia.ro (I.V.G.); 3 National Institute for Electrical Engineering, Advanced Research (INCDIE ICPE CA), 313 Splaiul Unirii, 030138 Bucharest, Romania; 4 National Research and Development Institute for Industrial Ecology-ECOIND, 57-73 Drumul Podu Dambovitei, District 6, 060652 Bucharest, Romania; costel.bumbac@incdecoind.ro (C.B.); ioana.ionescu@incdecoind.ro (I.A.I.); 5 IMT Bucharest, National Institute for Research and Development in Microtechnologies, 126A Erou Iancu Nicolae, 077190 Voluntari, Romania; bogdan.serban@imt.ro (B.-C.S.); octavian.buiu@imt.ro (O.B.)

**Keywords:** microalgae biomass, *Chlorella vulgaris*, SBS (styrene butadiene styrene), polymer blends, biodegradation

## Abstract

Bio-fillers are intensively studied for advanced polymer composite circular design and production. In this context, the algal biomass may be considered an important and relatively low-cost resource, when harvested as a by-product from wastewater treatment plants. The biomass of the algal species *Chlorella vulgaris* is frequently used in this type of environmental process, and its macro constituents’ composition ranges from around 15–25% carbohydrates, 10–20% lipids, and 50–60% proteins. Poly (styrene-butadiene-styrene) (SBS) copolymers have a matrix composed of glassy polystyrene domains connected by flexible polybutadiene segments. Although the physical-mechanical properties of SBS copolymers recommend them for many industrial applications, they have the drawback of low biodegradability. This study aimed to assess the aerobic biodegradability of polymer composites by integrating biomass from *Chlorella vulgaris* at varying mass percentages of 5, 10, and 20% into SBS copolymer composites. Biodegradation tests were conducted under industrial composting conditions (58 °C and 50% relative humidity) for 180 days. The biodegradability of materials was evaluated by measuring the CO_2_ produced in each vessel during the study period. Potential correlations between the amount of carbon dioxide released and the percentage of biomass added to the polymer matrix were examined. Structural and morphological changes were assessed using Fourier Transform infrared spectroscopy (FTIR), thermal analysis (DSC), and scanning electron microscopy (SEM). Physical and chemical testing revealed a decrease in sample density after the industrial composting test, along with noticeable changes in melt flow index (MFI). The observed physical and chemical changes, coupled with FTIR, SEM, and DSC data, indicate increased cross-linking and higher porosity in biodegraded polymer structures with higher biomass content. This behavior is likely due to the formation of cross-linked connections between polymer chains and polypeptide chains resulting from protein degradation, enhancing connections between polystyrene units facilitated by peptide bonds with the benzene units of the styrene blocks within the polymer matrix.

## 1. Introduction

The mismanagement of plastic waste and the resulting pollution have become significant environmental concerns in the past years [1,2]. The pollution with plastic waste, including microplastics, poses threats to biodiversity, ecosystem health, and human well-being and makes plastics and plastic waste an urgent environmental issue [3,4]. By incorporating bio-fillers derived from renewable sources like microalgae, the biodegradability of polymers can be improved, reducing their environmental impact and promoting a more sustainable approach to polymer materials [5,6,7]. These bio-based composites may have improved biodegradability and recyclability, reducing the persistence of plastic waste in the environment [8,9,10]. Furthermore, the use of bio-fillers may enhance the mechanical and functional properties of polymers, expanding their potential applications [11,12,13]. By incorporating bioactive compounds like microalgae biomass or other bio-fillers, the resulting materials may offer additional benefits, such as antimicrobial properties or antioxidant activity [14,15,16,17].

Microalgae have gained attention in recent years due to their potential as a sustainable and renewable resource for various applications, including in the field of materials science, and are regarded as third-generation biomass feedstock due to their numerous benefits [18]. The categorization of feedstocks into generations is based on their sustainability, resource efficiency, and potential for commercialization [19,20,21]. Microalgae can be grown on non-arable land, such as in ponds, bioreactors, or even wastewater treatment facilities, reducing the competition for agricultural land with high-biomass productivity, making them attractive for biofuel, bio-based products, and environmental applications. Additionally, microalgae can be cultivated using carbon dioxide from industrial processes, providing a potential solution for nutrient recycling and carbon capture, and is thus considered for sustainable resources [22,23,24].

Utilizing microalgae as a bio-filler can contribute to reducing the reliance on non-renewable resources and mitigate environmental impacts associated with traditional fillers. Microalgae can be mixed with various petroleum-based plastics such as HDPE (High-Density Polyethylene), LDPE (Low-Density Polyethylene), PVC (Polyvinyl Chloride), PP (Polypropylene), or bio-based polymers such as polyhydroxyalkanoates (PHA) and PLA (Polylactic Acid), which can effectively extend the durability of plastic material obtained with/from microalgae, and improve its mechanical properties [25,26,27]. Yang et al. performed a study with microalgae *Chlorella* sp. HS2 as a new resource of biomass to develop microalgae-based bioplastic materials by mixing it with LDPE, poly(ethylene–vinyl acetate) (EVA), and PLA. The results of the study showed that *Chlorella* sp. HS2 biomass disperses in the polymer to the micrometer scale without additional chemical treatment and is a promising biomass filler for bioplastic production [7]. 

Microalgae biomass is a biodegradable material, and it is reasonably presumable that incorporating it in polymer matrices may enhance the biodegradation properties of resulting composites. This is particularly useful for developing eco-friendly and sustainable polymer materials [28,29,30].

The *Chlorella vulgaris* biomass is rich in proteins, essential amino acids, carbohydrates, vitamins, minerals, and other bioactive compounds with the macro constituents composition (*w*/*w*) of 15–25% carbohydrates, 10–20% lipids, and 40–60% proteins [31,32]. *Chlorella vulgaris* is one of the most well-known microalgae species. Due to its versatility and potential applications, *Chlorella vulgaris* has been extensively studied in domains like food and nutrition, bioenergy, bioremediation, and biotechnology. Research scientists are still exploring its full potential and are optimizing the cultivation methods to effectively harness its benefits [23,33,34]. *Chlorella* has a relatively small cell size and high protein and carbohydrate composition, which favors the processing of polymers into films and fibers where the limiting factor is the particle size. In addition, it is considered suitable for bioplastic conversion without a pretreatment step, thus leading to cost-effective, large-scale production and reduced waste production [35]. 

Poly(styrene-butadiene-styrene) (SBS) copolymers are widely used in various industrial applications due to their desirable physical and mechanical properties. However, one of the drawbacks of SBS copolymers is their low biodegradability [36,37]. SBS copolymers consist of glassy polystyrene (PS) domains connected by flexible polybutadiene (PB) segments. The glassy PS domains provide strength, rigidity, and thermal stability to the copolymer, while the PB segments offer flexibility and elastomeric properties. This unique combination of properties makes SBS copolymers suitable for applications such as adhesives, sealants, coatings, and modifiers for plastics [38]. Unfortunately, the presence of the non-biodegradable polystyrene component in SBS copolymers significantly hinders their biodegradability. Polystyrene is a synthetic polymer that cannot be easily broken down by natural microorganisms in the environment. As a result, SBS copolymers persist in the environment for a long time, contributing to undesirable plastic pollution. To address the issue of low biodegradability, research scientists are actively working on developing alternative SBS composites. In this respect, some approaches include the incorporation of biodegradable components into the copolymer structure, modifying the copolymer through chemical or enzymatic degradation methods, or seeking environmentally friendly disposal methods such as recycling or energy recovery [37,39,40].

The present study reports original data on the influence of *Chlorella vulgaris* biomass incorporated into a styrene-butadiene-styrene composite copolymer in different mass percentages (5, 10, and 20%) on the aerobic biodegradability of newly resulted bio-composite polymers, in industrial composting conditions (58 °C, 50% RH/relative humidity). In addition, structural and morphological changes of the tested bio-composite samples during the biodegradation process are presented. Comparative data for studied SBS polymer compounds with and without added biomass are also provided. 

The results of this study provide insights into the effectiveness of incorporating *Chlorella vulgaris* biomass into enhancing the biodegradability of the SBS composites. If successful, this approach could have potential applications in various industries, including packaging, construction, and automotive, where SBS composites are commonly used.

## 2. Materials and Methods

### 2.1. Polymer Bio-Composites Preparation

For the study, a composite base–polymer was prepared containing 25% (*w*/*w*) linear SBS (30% styrene) copolymer, 50% (*w*/*w*) branched SBS (40% styrene) copolymer, and 25% (*w*/*w*) paraffin oil. The mass ratio of linear SBS to branched SBS was 1:2 to ensure that the paraffin oil could be absorbed up to 25% (*w*/*w*) without exudation, which was determined to be the optimal percentage for incorporating biomass and producing microalgae SBS samples. This recipe was established following preliminary tests aimed at maximizing the incorporation of algal biomass. Lowering the percentage of paraffin oil resulted in extrusion machine clogging when higher proportions of algal biomass (up to 20%) were added. Additionally, it was observed that paraffin oil did not properly integrate into the polymer matrix when the content exceeded 25%. Branched polymers exhibit a greater absorption of paraffin oil; however, employing SBS branched polymer alone led to a blend that failed to homogeneously incorporate up to 20% *Chlorella vulgaris* biomass during extrusion. Consequently, 25% of linear SBS was introduced to enhance the flowability of the polymer blend. In addition, preliminary tests demonstrated that a paraffin oil percentage lower than 25% hindered the incorporation of algal biomass at the intended proportion (up to 20% *w*/*w*). Moreover, exceeding 25% paraffin oil content resulted in improper integration into the polymer mass. Furthermore, the inclusion of curing agents like maleic anhydride in the base–polymer matrix was avoided.

The microalgal biomass of *Chlorella vulgaris* (powder with 5 ± 0.5% moisture) was then incorporated in the base–polymer matrix so that the biogenic content in the final bio-composite polymer was of 5, 10, and 20% (*w*/*w*). The incorporation of the biomass powders was facilitated by the relatively high percentage of paraffin oil in the base–polymer. Materials suppliers were, respectively for SBS-type copolymers, LCY Grit Corporation (Taiwan), Apar Ltd., Mumbai, India for the paraffin oil, and Hyperici Pharm SRL, Targoviste, Romania for *Chlorella* powder.

### 2.2. Aerobic Biodegradation Setup

The installation employed for the biodegradation tests was developed in-house, following the indications of ISO 14855-1/2012 standard procedure [41], with several necessary modifications according to laboratory specificity. Thus, each climatic chamber (Haida International, Dongguan City, Guangdong Province, China) held eight tightly covered glass cylindrical vessels with individual test samples, blank samples, and control samples, respectively. A measured quantity of a 100 g sample was placed in each vessel together with 600 g vermiculite (d.w.). The latter was previously activated according to the standard ISO procedure. The overall moisture of this mixture was maintained at 50% ± 2% during the whole experiment by weekly checking of the overall weight, and adding deionized water when needed. Each testing vessel constantly received dry and CO_2_-free air on the inlet circuit, at a flow rate of 2 L/minute (compressor KA5-ESM5, air dryer EDX9-CC1030394, Garden Denver, Parma, Italy). The evolved gases during sample biodegradation were conducted via silicon tubes with unidirectional valves to the 500 mL glass gas-washers filled with NaOH solution with a known concentration (optimal concentration for our tests was concluded to be 0.5 N). The decrease in alkaline solution concentration was monitored by titrations (T5 potentiometric titration system with InMotion Flex autosampler, Mettler Toledo, Greifensee, Switzerland) with HCl 0.1 N solution. Samples of the sodium hydroxide solutions corresponding to each biodegradation vessel were periodically collected (10 mL, triplicate measurements) from gas washers, and according to data obtained on the respective day, a decision was made if there was a need to change the *receiving* NaOH 0.5 N solution with a freshly prepared one. This action was related to ensuring a reasonable available NaOH quantity to react with potentially evolved CO_2_ from the sample degradation processes of each sample. It is worth mentioning that the final calculations of CO_2_ that evolved from each vessel took into consideration the following data: titer of as-prepared NaOH 0.5 N solution, titer of as-prepared HCl 0.1 N solutions, and the amount of hydroxide volume sampled for potentiometric titrations.

**Figure 1:**
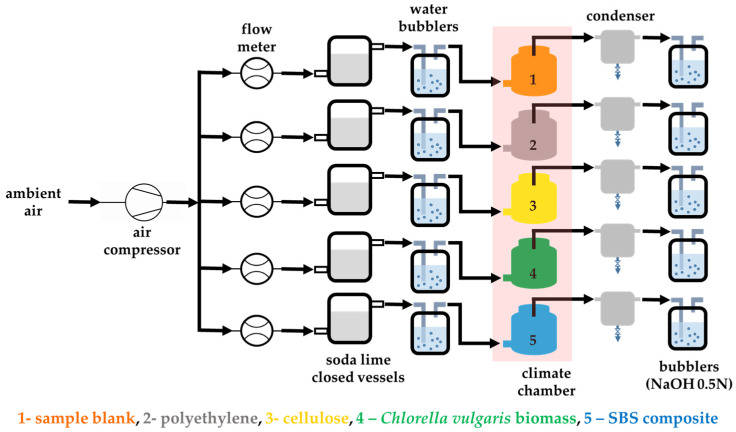
Schematic representation of the laboratory biodegradation testing installation.

As indicated in Figure 1, the aerobic biodegradation experimental installation used was modified compared to the provisions of ISO 14855 standard [41]; these changes were performed after preliminary studies, for the easiness of long-term operation (up to six months). On the one hand, for the inlet circuit, to obtain CO_2_-free air, the sodium hydroxide solution (liquid) was replaced by soda-lime (solid) for the following reasons: the reference “CO_2_-free” baseline may be quasi-instantly obtained in this setup, and also it may be maintained to a value of 300 ± 20 ppm for the whole test duration (no need to change the soda-lime in the 6 months’ timeframe of the experiments). On the other hand, for the gas outlet circuit, decanters were mounted in the proximity of the climatic chambers, to reduce the amount of water arriving in the “receiving” NaOH solution, thus leading to undesirable dilution, which should then be considered for the released CO_2_ from bio-degradation process calculations. The use of 0.5 N sodium hydroxide solution was established as optimal for absorption of the CO_2_ that resulted from the bioreactors. The *receiving* NaOH solution in the bubblers was changed when its initial concentration was halved.

The samples were codified as: SBSC (control sample of styrene-butadiene-styrene composite), SBSCh5, SBSCh10, and SBSCh20 (composites with 5%, 10%, and 20% *Chlorella vulgaris* biomass content).

### 2.3. Structural and Morphological Characterization Techniques

▪ Physical-mechanical testing

The evolution of flowability and specific gravity of the bio-composite polymer materials after two biodegradation periods (100 days and 180 days) in the previously described conditions was followed. The testing to evaluate the impact resistance was carried out using the testing machine for determining the shock resistance is a pendulum hammer Haida HD-R802-2 Plastic Chary & Izod Impact Tester (Haida International Dongguan City, China) for unnotched samples following the described work procedure in ISO standard 180:2023 [42]. 

The testing eprouvettes were conditioned according to EN ISO 291 [43]. The melting flow index was measured (standard method SR ISO 1133:2022 [44]) with a Melt Flow Index Tester (Haida Plastic Melt Flow Index Testing Machine, Dongguan, China), while specific gravity (SR ISO 2781:2018 [45]) was measured with the VF4601 density kit mounted on a SECURA225D-1CEU balance (Sartorius, Göttingen, Germany). 

To elucidate the impact of microalgae biomass addition to the polymer, impact tests were conducted for the non-biodegraded samples and compared with tensile strength values obtained from non-biodegraded samples. Unfortunately, testing the resistance of biodegraded collected samples proved unfeasible, as the recovered polymer pellets underwent drying as outlined in the experimental procedure. Despite various attempts to procure suitable samples for tensile strength and Izod impact tests while minimizing sample quantity, none were successful. Biodegraded samples, particularly those containing microalgae biomass, exhibited reduced flowability, as indicated by MFI tests. Elevated temperatures were necessary for their processing, leading to a major drawback: the formation of gas bubbles within the material, likely due to reactions among biodegradation intermediates.

▪ Fourier Transform Infrared spectroscopy (FTIR)

Potential changes in the molecular structure of polymer bio-composites, in the biodegradation conditions as-described, and versus those of the base–polymer matrix were evaluated through Fourier transform infrared (FTIR) spectroscopy. 

Data acquisition was performed with a Vertex 80 infrared spectrometer (Bruker, Karlsruhe, Germany) with an attenuated total reflection (ATR) system, and solid samples were used with no need for further preparation. The scanning parameters were as follows: the wavenumbers ranged 4000 cm^−1^ to 400 cm^−1^, the selected spectral resolution was 4 cm^−1^, 32 scans per sample were recorded, and the average spectrum was used for data interpretation. Control samples (base–polymer matrix SBSC), and products after the two studied biodegradation times were scanned.

▪ Scanning Electronic Microscopy (SEM)

Morphological investigations of composite polymers containing algal biomass in different mass ratios were performed by electronic scanning microscopy using the system SEM-Quanta FEG 250 (Thermo Fischer Scientific, Waltham, MA, USA) in secondary electron mode using the Everhart-Thornley Detector. Images were obtained on freshly cut, 2-mm-thick samples mounted on microscopic stubs, after fully covering them with carbon bands to ensure the electrical conductivity needed for SEM measurements. Morphology comparisons of bio-composite eprouvettes after 100 and 180 days of aerobic biodegradation tests versus their initial surface condition were discussed.

▪ Thermal Analysis—Differential Scanning Calorimetry (DSC)

The characterization of polymer bio-composites and control samples (base–polymer matrix) was performed using the DSC3-StarE thermal analysis system (Mettler Toledo, Greifensee, Switzerland). For this purpose, hermetically sealed aluminum pans of 40 µL were filled with 10 ± 1 mg of the studied sample, and tests were run in the temperature range of 30 to 300 °C, at a heating rate of 10 °C/min. An empty and sealed 40 µL aluminum pan was used as a reference in all DSC tests.

## 3. Results

### 3.1. Industrial Composting Test

**Figure 2:**
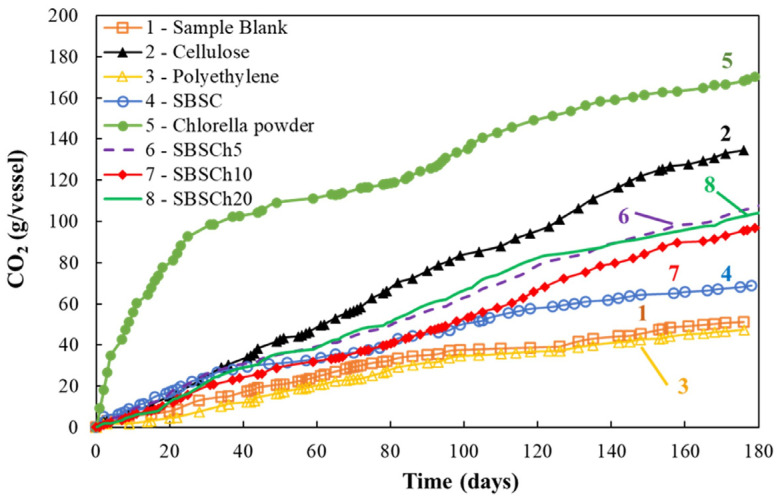
Evolution of CO_2_ mass (g/vessel) trapped in the NaOH 0.5 N solution in the biodegradation test (industrial composting).

Figure 2 shows the variation in the mass of carbon dioxide trapped in the 0.5 N NaOH solution from the bubblers after the titration of the solution.

The total mass of CO_2_ that was absorbed in the bubbles was calculated according to Equation (2), where ${m_{{CO}_{2}}}_{i}$ is the carbon dioxide mass calculated according to Equation (1) between two titration points “*i*” and “*i* + 1”. The values presented for $c_{i}$ and $c_{i+1}$ represent the concentrations of sodium hydroxide solutions at the titration points “*i*” and “*i* + 1”, whereas the volumes $V_{i}$ and $V_{i+1}$ are the volumes of solutions in bubblers before titration.

(1) $${m_{{CO}_{2}}}_{i+1}={m_{{CO}_{2}}}_{i}+\left(c_{i}\cdot V_{i}-c_{i+1}\cdot V_{i+1}\right)$$

(2) $${m_{{CO}_{2}}}_{total}=\sum_{i=1}^{n}{m_{{CO}_{2}}}_{i}$$

**Figure 3:**
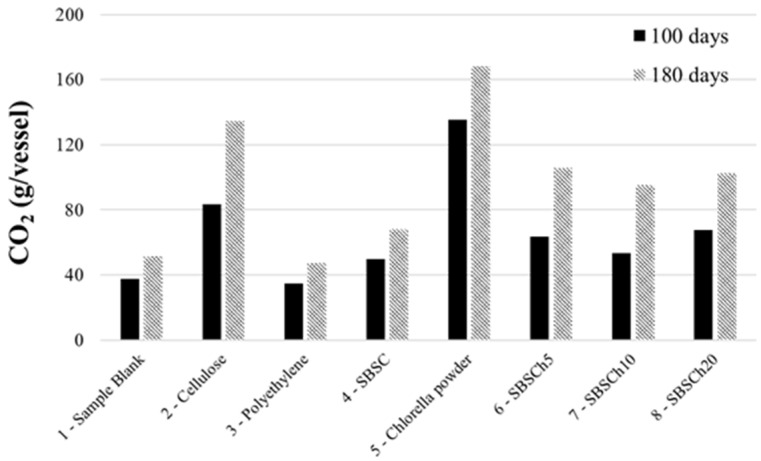
Comparative values of CO_2_ mass (g/vessel) absorbed in the industrial composting biodegradation test: after 100 days, and 180 days.

The evolution of the CO_2_ released from the bioreactors shows that polyethylene (the negative control) produced a lower amount of CO_2_ than the sample blank (the bioreactor that contained only the compost support used for the biodegradation study). Among the curves presented in Figure 2, the evolution of the mass of CO_2_ trapped from the bioreactor with *Chlorella vulgaris* biomass stood out compared with the other bioreactors. The amount of CO_2_ released was even much higher than the positive control that is the bioreactor with the same mass of cellulose. The explanation of this behavior is based on the specificity of *Chlorella* biomass, which has high starch content and other macroconstituents that could break down easier, thus supporting the development of microorganisms involved in the biodegradation process [46]. The differences are even more obvious in the graph that shows the comparison of the values of the amounts of carbon dioxide trapped in the bubblers at 100 days and 180 days (Figure 3).

Analyzing the curves presenting the carbon dioxide mass released from the bioreactors with SBS composites, it was observed that up to 100 days, small differences are recorded between studied materials, regardless of whether they have biomass incorporated or not. After 100 days, it was observed that the amount of CO_2_ released by the polymer composites that contained biomass becomes higher compared to the mass of CO_2_ released by the SBS composite, indicating that the polymer that did not contain microalgae biomass had reached the moment when the material did not degrade. Meanwhile, the materials that contained *Chlorella* biomass continued to release carbon dioxide in a higher quantity than the control sample (SBSC), thus indicating continuation of the biodegradation process of these materials.

The data presented in Figure 2 and Figure 3 suggest that the microalgae not only serves as a biodegradable filler, but may also actively improve the compostability of the polymer by providing nutrients or other compounds that facilitate the growth and the activity of decomposing microorganisms that can grow and adapt within the SBS polymer matrix.

### 3.2. Physical Properties Testing of Polymer Composites

**Figure 4:**
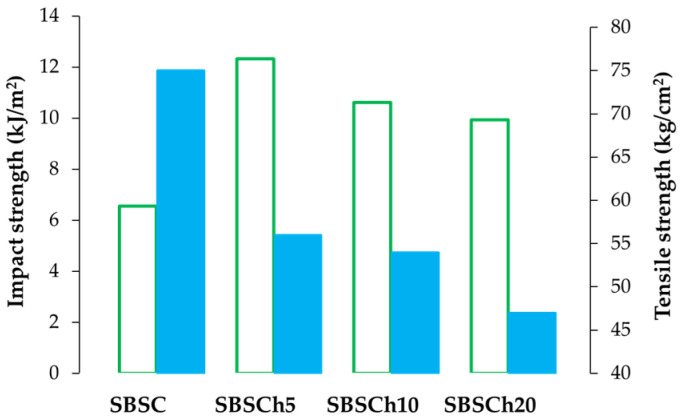
Compared values of impact strength (green empty bar) with tensile strength (blue bar) for SBS composites with different loaded microalgae biomass content.

When considering the resistance to impact and tension, it becomes apparent that SBS composite samples with microalgae exhibit distinct behavior compared to polymer samples without microalgae. Analysis from Izod impact testing reveals that the SBS composite without added microalgae displays lower impact resistance than the polymer with microalgae, despite having higher tensile strength values. Additionally, a trend was observed where both impact strength and tensile strength decrease when comparing samples with 5%, 10%, and 20% *Chlorella biomass* added to the polymer matrix (Figure 4).

**Figure 5:**
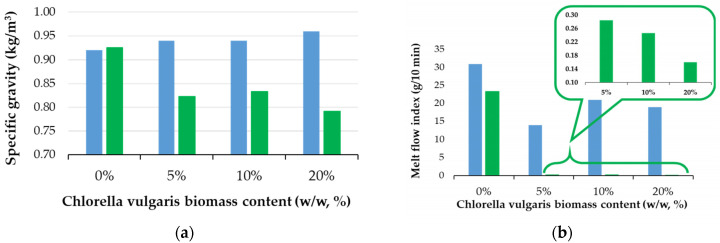
Physical properties of polymer composites: (**a**) specific gravity and (**b**) melt flow index for initial samples (blue bar) compared with samples after 180 days of an industrial composting biodegradation test (green bar).

The observed changes in specific gravity and melt flow index (MFI) presented in Figure 5a,b offer interesting insights into the physical and chemical alterations that polymer composites undergo during the biodegradation process, particularly with the inclusion of *Chlorella vulgaris* biomass.

The initial increase in the specific gravity for composites with microalgae compared to those without incorporated biomass (blue bars in Figure 5a) can be attributed to the denser nature of the *Chlorella biomass* compared to the base–polymer matrix. Microalgae, being rich in cellular components like carbohydrates, proteins, and lipids, can increase the density of the composite material [46].

After undergoing the industrial composting biodegradation test, the reduction in specific gravity for the composites, especially those with 20% biomass content, is noteworthy. The specific gravity reduction from 0.95 kg/m^3^ to 0.80 kg/m^3^ could be indicative of several processes that involve the biodegradation of *Chlorella* biomass and polymer matrix, leading to the formation of pores in the material, as the biomass and the polymer are consumed by microorganisms. Subsequently, it will be expected that the higher the biomass content, the lower the density of biodegraded material will be, because the biomass degrades faster as shown in Figure 2. Furthermore, the polymer matrix’s degradation, which, while slower, contributes to the overall modification in density. The presence of *Chlorella* might accelerate this process by either acting as a site for microbial colonization, or/and by altering the chemical structure of the base–polymer to make it more susceptible to microbial attack.

Changes in melt flow index (MFI) could indicate a change in the polymer’s molecular weight distribution, usually due to breakdown or chain scission events [47]. Figure 5b presents the evolution of the melt flow index (MFI) for the SBS composites before and after the biodegradation test, and thus adds useful information for the understanding of how *Chlorella* biomass may affect the polymer’s properties while biodegrading under industrial composting conditions. A decrease in MFI generally indicates an increase in the material’s viscosity, suggesting that polymer chains are longer or more entangled.

Experimental findings, as indicated in Figure 5b, are discussed herein from the perspective of the biodegradation processes’ influence on the MFI of studied materials containing different biomass loadings. 

Figure 5b shows a predictable decrease in MFI for all samples after a 6-month biodegradation test (green bars), when compared with initial MFI values (blue bars). The significant reduction in MFI after biodegradation, especially for the biomass-loaded samples (to values lower than 0.3 g/10 min), is particularly noteworthy. The SBSC composite MFI decrease from 31.0 ± 0.5 g/10 min to about 23.3 ± 0.6 g/10 min shows that the material changes, likely due to a partial biodegradation of the polymer matrix itself. A valuable experimental finding is that the biomass-loaded samples exhibit MFIs after a biodegradation close to zero, and this suggests a profound transformation of the material’s physical structure and chemistry in the industrial composting conditions. 

This significant MFI reduction could be due to several factors. With regards to this significant reduction in MFI after the 6-month biodegradation time, the following factors could be considered as an explanation.

- Cross-linking within the material between degraded molecules of polymer and short-chain molecules resulting from the biodegradation of biomass, can act as a bridge between polymer branches, leaving behind a more complex, less flowable residue. The presence of *Chlorella* biomass could facilitate a network structure that becomes highly rigid or entangled after partial degradation, significantly restricting flow [12,48].
- A loss of plasticity due to the biodegradation process that might break down components of the polymer matrix that contribute to its plasticity, leaving behind a material that is much more rigid. The flexibility of the SBS polymer chain is due to the polybutadiene segments that have unsaturated bonds susceptible to reaction with chemical species from the biodegradation media [12].
- The formation of pores in the polymer matrix, especially in biomass-loaded samples, might create a porous, sponge-like structure that does not flow under MFI testing conditions. This explanation is consistent with the specific gravity test, which shows a decrease in the specific gravity for the biomass-loaded samples.

### 3.3. FTIR Spectroscopy

**Figure 6:**
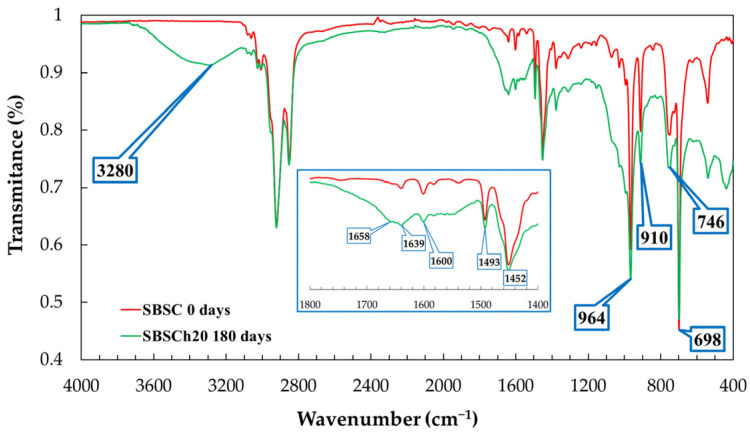
Infrared spectra of SBSC (initial, red line) compared with infrared spectra of SBSCh20 (after 180 days biodegradation test, green line).

Figure 6 shows the infrared absorption spectrum of the SBSC material (control sample) that was not subjected to the biodegradation test, and of the SBSCh20 sample after 180 days of industrial composting biodegradation testing. The two spectra were chosen for comparison because the spectra of the other samples containing biomass are similar, and have absorption band intensities between the absorption bands shown in Figure 6.

**Figure 7:**
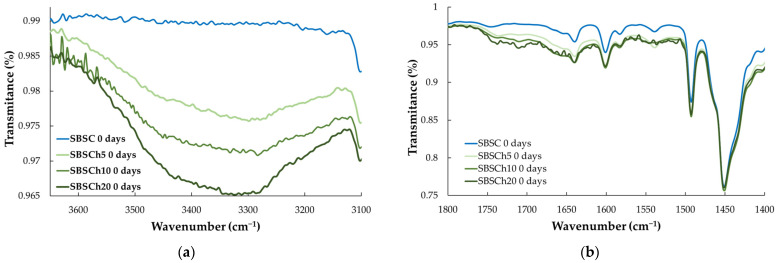
Infrared spectra of SBS-based composites before biodegradation in the region: (**a**) 3600–3100 cm^−1^, and (**b**) 1800–1400 cm^−1^.

The initial samples, not subjected to the biodegradability test, show similar spectra, with small variations in the 3600–3200 cm^−1^ domain, where the absorption bands related to O–H and N–H stretching vibrations (Figure 7a), which may be observed. In the 1800–1400 cm^−1^ wavenumber region, the infrared spectrum is similar for samples not subjected to biodegradation (Figure 7b), while the biodegraded samples present a broad band with maxima at 1550, 1639, and 1658 cm^−1^ (Figure 6 inset). The appearance of these vibration bands in the spectrum of biodegraded samples can be correlated with the formation of peptide bonds in the polymeric material [49,50]. The absorption bands at 746, 910, and 964 cm^−1^ are characteristic of cis-1,4, 1,2, and trans-1,4 butadiene units, respectively [51]. 

The absorption peaks that appear in the 1000–650 cm^−1^ region of the IR spectra are assigned to C–H bending vibrations where C has sp^2^ hybridization (=C–H bending). The band that appears at 746 cm^−1^ is correlated with the presence of monosubstituted alkenes, while the band at 910 cm^−1^ shows the vibrations of disubstituted alkenes. The band at 1450 cm^−1^ is correlated with C–H (C sp^3^) scissoring vibrations, while -C–H stretching vibrations of C sp^3^ arise at 2920 and 2850 cm^−1^ [52].

The band at 964 cm^−1^ is indicative of the 1,4-trans structure of polybutadiene. The 1,4-trans configuration refers to the spatial arrangement of the substituents on the butadiene monomer units within the polymer chain. The presence of this band and its intensity are correlated with polybutadiene content within the SBS copolymer. The assumption that the content of the 1,4-trans structure in different types of SBS copolymers is very close allows for a simplified comparison and quantification. The absorption band at 698 cm^−1^ is characteristic of monosubstituted benzene rings, which is a key structural component of polystyrene (PS). 

The use of the ratio of absorbance at 964 cm^−1^ relative to 698 cm^−1^ provides a method to quantify the polybutadiene/polystyrene ratio in the copolymer. The absorbance ratio becomes a proxy for understanding the balance between the rubbery characteristics imparted by polybutadiene and the rigid, glassy nature of polystyrene within the SBS copolymer [53]. 

**Figure 8:**
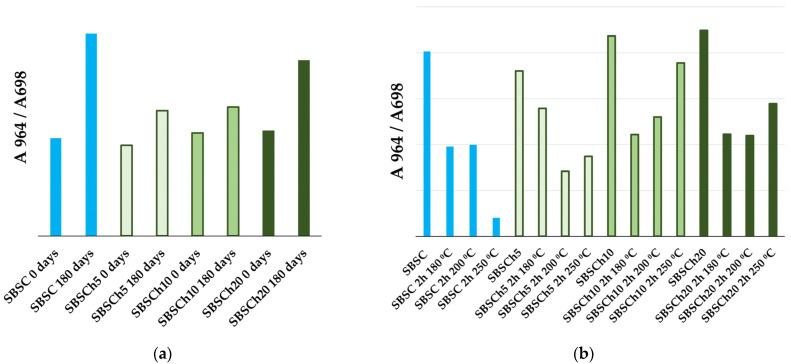
Comparison of absorbance ratios A 964 cm^−1^/A 698 cm^−1^ calculated for initial samples (blue bars) that undergo (**a**) a biodegradation test, and, respectively, (**b**) a thermal degradation test (green bars).

The variation in the ratio between the absorbances of the absorption peaks from 964 cm^−1^ and 698 cm^−1^ indicates two different processes that may occur in the materials tested in the two comparative tests. In the case of the biodegradation test, the ratios of absorbances increase for all samples indicating an increase in the polybutadiene content or a decrease in the number of monosubstituted benzene nuclei in the polymer structure (Figure 8a). By comparison, Figure 8b shows the ratio of the polymer that has undergone thermal degradation and it is observed that the ratios record the reduction in values with the heating of the material, indicating the decrease in the content of polybutadiene in relation to that of polystyrene (Figure 8b). These variations can be attributed to two different cross-linking mechanisms, as will be explained in the paragraphs below.

The absorption bands 1493 cm^−1^ and 1600 cm^−1^ are specific to C–C stretching aromatic ring mode vibrations. Most infrared spectrometers are sold with a polystyrene sample, and the position of this peak at 1600 cm^−1^ has been used for decades as a wavenumber standard [54]. In the case of our materials, it is observed that the position of the two peaks remains unchanged, but the intensity of the band at 1600 cm^−1^ is influenced by the broad band that appears in the spectra of the biodegraded samples in the 1700–1600 cm^−1^ wavenumber range. The influence of the broad band in the 1700–1600 cm^−1^ range on the intensity of the 1600 cm^−1^ band, particularly in biodegraded samples, could compromise the reliability of using the latter for comparative analysis. Therefore, it was decided to use the absorption band at 1493 cm^−1^ as the reference band for comparing the spectra, because the position, width, and intensity remained approximately the same for all tested samples, and thus it provides a more stable reference point. 

**Figure 9:**
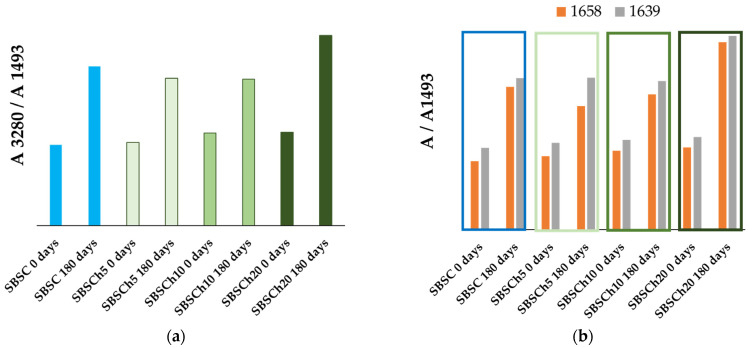
Compared values of absorbance ratios for studied samples before (blue bars—0 days) and after (green bars—180 days) the biodegradation test, calculated as absorbance values at 3280 cm^−1^ (**a**), and respectively, at 1658 (orange bar) and 1639 (gray bar) cm^−1^ (**b**), relative to absorbances measured at 1493 cm^−1^.

Figure 9a shows the ratio of the absorbance measured at 3280 cm^−1^ relative to the absorbance measured at 1493 cm^−1^. It can be observed, in all cases, that the biodegradation test leads to materials with a broad absorption band with a maximum at 3280 cm^−1^ (specific to O–H and N–H stretching vibrations) with higher intensity than the composite samples not subjected to the biodegradation test (0 days). The maximum ratio was recorded for the polymer composite samples that contained 20% *Chlorella vulgaris* biomass incorporated. Furthermore, the absorbance ratios measured at 1658 cm^−1^ and 1639 cm^−1^ relative to the absorbance of the 1493 cm^−1^ reference band are higher for the biodegraded samples in all cases, indicating the possibility of the formation of peptide bonds in the structure of the material.

### 3.4. SEM Analysis

**Figure 10:**
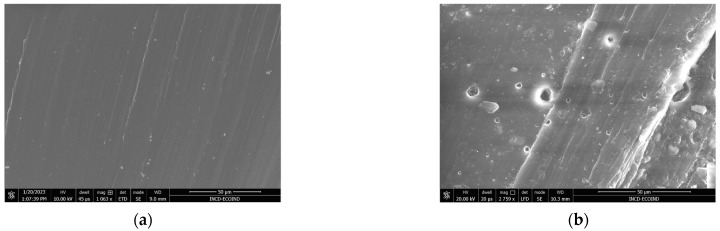

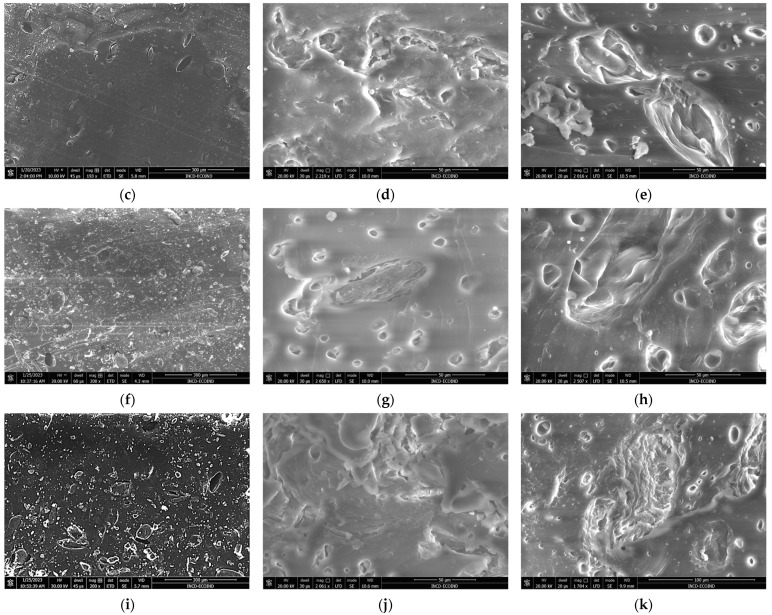
SEM images of polymer SBS composites, (**a**) SBSC initial sample, (**b**) SBSC after 180 days and SBS *Chlorella* composites, (**c**) SBSCh5 initial sample, (**d**) SBSCh5 after 100 days, (**e**) SBSCh5 after 180 days, (**f**) SBSCh10 initial sample, (**g**) SBSCh10 after 100 days, (**h**) SBSCh10 after 180 days, (**i**) SBSCh20 initial sample, (**j**) SBSCh20 after 100 days, and (**k**) SBSCh20 after 180 days.

The images presented in Figure 10a–k show the surface morphology of the composite polymer materials before and after the biodegradation test. The images were taken for the section of samples that have been sectioned just before measurement. 

Upon incorporating the biomass particles into the polymer matrix, it was noticed that a fraction of the algal biomass particles become trapped within cavities, rendering them immobile (as illustrated in Figure 10c–k). Scanning Electron Microscope (SEM) imagery reveals micrometric imperfections, both in terms of number and dimension, which escalate as the proportion of embedded biomass rises.

Furthermore, it was observed that the biodegradation test of polymer composites leads to the degradation of the base–polymer network. The phenomenon is also visible in the case of the SBSC control sample to a lesser extent (Figure 10a,b), while the presence of *Chlorella vulgaris* biomass influenced the behavior of the material. Thus, it can be seen that composite materials with biomass changed the appearance of craters and cracks in the polymer matrix.

The presence of cracks and holes is indicative of surface degradation, which is a common initial sign of biodegradation in polymers. This suggests that the microalgae, either directly through its biological activity or indirectly by altering the local environment (e.g., pH, moisture), has contributed to the breakdown of the polymer’s surface. This could be due to the secretion of enzymes or other bioactive compounds by the microalgae that either attack the polymer chains or catalyze attack, leading to the observed physical alterations.

### 3.5. DSC Analysis

**Figure 11:**
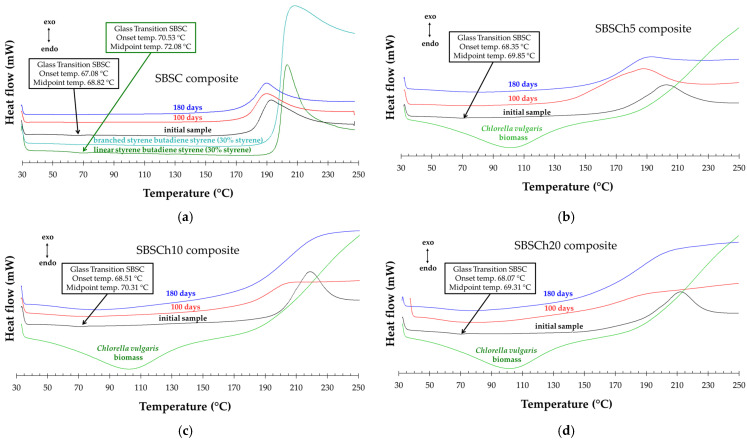
DSC curves of tested polymers recorded for samples heated from 0–300 °C at 10 K min^−1^ heating rate: (**a**) SBSC composite before and after 100 and 180 days compared with the SBS used to prepare the polymer blends, (**b**) SBSCh5 and *Chlorella vulgaris* powder, (**c**) SBSCh10 and *Chlorella vulgaris* powder, (**d**) SBSCh10 and *Chlorella vulgaris* powder.

The DSC analysis of the control samples, SBSC, (Figure 11a) showed that the new composite material had a glass transition similar to the linear SBS used for their preparation. It is noticeable that the temperature for the glass transition was lower with 3 °C in the composite than in the linear SBS component of the mixture. One may also observe that biodegraded SBSC samples had no glass transition. The observation that the control composite material exhibits a glass transition similar to that of the linear SBS, but lower, suggests that the addition of paraffin oil and the blend of linear and branched SBS influence the material’s flexibility and segmental mobility. It is known that paraffin oil acts as a plasticizer, reducing intermolecular forces and increasing the free volume, thereby lowering the glass transition temperature (Tg) [55].

The lack of a glass transition in samples subjected to biodegradation testing (constant temperature of 58 °C for the whole test duration) is particularly noteworthy. This experimental evidence related to Tg specific to styrene blocks from the composites (Figure 11a–d) indicates a change in the physical state of these blocks [56]. The disappearance of Tg is linked to the increased amorphousness of the styrene blocks. This could occur due to several factors, one of which could be the transformation of the monosubstituted benzene units within the composite. The transformation of monosubstituted benzene units could disrupt the regular packing of polymer chains, leading to amorphous styrene blocks. 

**Figure 12:**
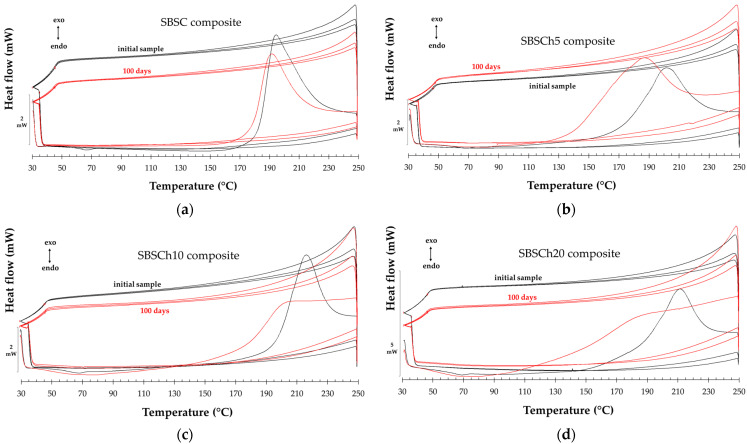
Comparative DSC thermograms of studied polymer composites without (**a**) and with incorporated *Chlorella vulgaris* biomass percentages of (**b**) 5%, (**c**) 10%, and (**d**) 20%, recorded on pristine samples (black lines), and on samples after 100 days of biodegradation (red lines), in dynamic thermal regime with 3 heating–cooling cycles, in the temperature range 30–300 °C, at a rate of 10 °C/min.

Figure 12 presents the DSC curves for heating–cooling in 3 cycles for initial samples and samples collected after 100 days of biodegradation in industrial composting conditions as described by ISO 14855 [41]. An experimental finding noticeable in this figure is that the exothermal effect recorded for all samples in the first heating–cooling cycle disappears when re-heating. On the other hand, for the second and third cycles, there is a difference in the thermal effects values between heating and cooling of about 0.3 mW/mg for all studied samples.

The disappearance of the glass transition and the exothermic effect upon reheating cycles between 30 °C and 300 °C in SBS may be influenced by their thermal history or by the induction of irreversible changes in the polymer matrix. Repeated heating and cooling cycles may induce changes in polymer structure, including relaxation processes and molecular rearrangements, which may alter the observed thermal transitions in the first cycle of heating. In addition, the first heating cycle may induce irreversible changes in the SBS composite structure, leading to the disappearance of specific thermal transitions upon subsequent cycles. These irreversible changes could include structural relaxation, cross-linking, or degradation processes.

**Figure 13:**
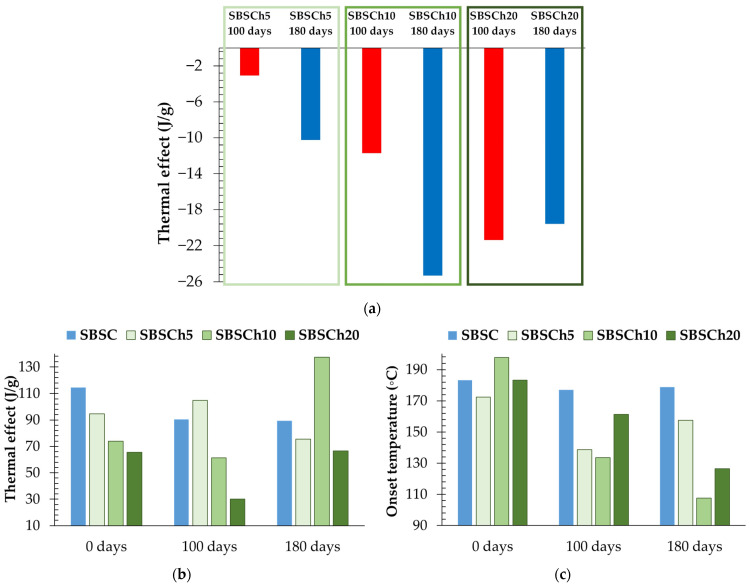
Representation of (**a**) normalized endothermal effect (J/g) registered in the 50–110 °C domain, (**b**) normalized exothermal effect (J/g), and (**c**) onset temperature variations in the exothermal effect, observed in the first cycle of heating for SBS composites filled with *Chlorella vulgaris* biomass.

In the first cycle of heating, a large endothermal effect between 50 °C (onset temperature) and 118 °C (end set temperature) was observed, which appeared only for the biodegraded samples that contained biomass. The representation of the endothermal effect normalized to sample weight is presented in Figure 13a. The variation in the endothermal effect is related to the biomass content in the sample. This behavior suggests changes in the SBS composites structure post-biodegradation. The large endothermal effect near 50 °C indicates heat absorption, which can be attributed to processes like desorption, sublimation, or dehydration. *Chlorella* biomass presents, also, a large endothermal effect, but the thermal effect appears in the 80–110 °C domain (near 100 °C, the boiling point of water) with a normalized value seven times higher than the SBSCh20 composite after 180 days of biodegradation in industrial composting conditions (Figure 11b–d). The differences observed could be explained by the desorption of water or other substances that accumulated within the polymer matrix during these tests. Biodegradation processes often generate by-products or break down polymer chains, leading to the release of trapped moisture or volatile compounds upon heating [57]. The larger negative effect observed in samples with higher biomass content, even after drying in the same conditions, suggests a correlation between biomass content and the endothermal effect. Higher biomass content may result in a greater retention of moisture or decomposition by-products within the composite, leading to enhanced endothermal effects upon heating.

The exothermal effect that appears in the first heating cycle (Figure 11a–d) becomes broader for the degraded samples compared with the pristine ones, while the effect is more pronounced in the case of samples filled with microalgae biomass. Figure 13 presents the evolution of the normalized-to-weight effect for all the studied samples. In the pristine samples, there is an evident connection between the biomass content and the thermal effect. For the biodegraded samples, the thermal effect has a broader shape, while there is no correlation between the normalized thermal effect and the biomass content in the composites after 100 days and 180 days.

The evolution of the exothermal effect onset temperature of the first cycle’s heating is presented in Figure 13c, which indicates that industrial composting testing has the effect of decreasing the onset temperature for all the samples. The lowest onset temperatures were recorded for SBSCh10 and SBSCh20 composites after 180 days of testing.

## 4. Discussion

The use of whole microalgae rather than extracted components from microalgal biomass was studied for blends with polyethylene (PE) and polypropylene (PP). So far, the incorporation of microalgae into polymers has primarily aimed at enhancing mechanical properties. However, a recent report underscores the stabilizing effects of microalgae in PP [58]. As reported by Mateescu et al., microalgal biomass acted as an agent to protect the degradation of PP, primarily caused by polyphenols in microalgae. Hossein Tafreshi et al. reported that the inclusion of *Chlorella vulgaris*, combined with polyethylene glycol (PEG), significantly enhanced resistance to gamma irradiation [59]. For manufacturers that only require low-temperature processing, water-soluble polyvinyl alcohol (PVA) could be a good option as it can be processed under the boiling temperature of water. In addition, the use of polyurethane with microalgae can take advantage of superior mechanical properties, although it requires a complex process, which possibly increases costs. This shows an interest in making bioplastics using microalgae, which is a good alternative to following the trend of eco-friendly materials [60].

The significant changes observed during the biodegradation experiment of SBS composites with *Chlorella vulgaris* provide information about structural and thermal transformations occurring within the polymer matrix and could be translated into a possible mechanism of polymer degradation. 

The observation that initial SBS composite samples without microalgae have lower impact resistance but higher tensile strength compared to those with microalgae suggests a trade-off between these properties. The higher impact resistance observed for microalgae-loaded polymers compared to the base–polymer matrix shows that microalgae particles may act as reinforcement within the polymer, dispersing energy more effectively upon impact and reducing the propagation of cracks. Furthermore, the decrease in tensile strength of the polymer without microalgae as the content of microalgae biomass increases could be correlated with the disruption of the molecular structure and intermolecular interactions of the polymer. This disruption can create defects, voids, or weak points within the material, reducing its overall strength when elongated.

This is because the microalgae biomass may not be fully compatible with the polymer matrix, leading to poor adhesion between the microalgae particles and the polymer. This lack of adhesion can result in stress concentrations at the interface between the polymer matrix and the microalgae particles, leading to a material showing microdefects with trapped biomass, as confirmed by SEM analysis.

The decrease in material density after 180 days of composting indicates structural changes within the SBS composites. Biodegradation processes likely lead to the breakdown of macroconstituents in the biomass, in the polymer chains, or the removal of molecular fragments, resulting in a less dense material. This is well correlated with the SEM images that present a material with a porous structure, the pores being larger in the case of samples with added biomass, after 180 days of composting. The dramatic decrease in MFI, for samples with biomass compared with SBSC composite, suggests increased molecular weight or chain entanglement within the polymer matrix post-biodegradation. This reduction in MFI indicates a decrease in the polymer’s flowability, which could result from cross-linking or increased polymer chain interactions. These findings are supported by FTIR and DSC analyses of the pristine samples, compared with the polymer composites collected after 100 and 180 days of biodegradation in industrial composting conditions. 

The infrared spectroscopy analysis, as indicated by the ratio of the absorbance A964/A698 (absorbances measured at 964 cm^−1^ and 698 cm^−1^), suggests, on one hand, a relative decrease in monosubstituted benzene units compared to butadiene from the base-polymer matrix. This change in composition could result from selective degradation of styrene blocks or preferential transformation of styrene-rich regions during biodegradation. On the other hand, the observed increase in IR absorbance relative to the reference peak of 1493 cm^−1^, at specific wavenumbers can be associated with peptide bond formation (3280 cm^−1^, 1658 cm^−1^, and 1639 cm^−1^) in the polymer structure post-biodegradation. This finding implies the involvement of microbial activity in polymer and biomass breakdown, potentially leading to the formation of cross-links or covalent bonds between polymer chains and the polypeptide units resulting in the process. This structural modification could contribute to changes in material properties such as mechanical strength, thermal stability, and degradation resistance.

The findings reported on the aromatic degradation of styrene align with the results presented in this study. Various microorganisms demonstrate the ability to undergo aerobic growth using styrene as their sole source of carbon and energy [12,61]. In aerobic conditions, SBS may undergo metabolism through the oxidation of the C=C bond in polybutadiene segments. However, previous studies have also reported the oxidation of the aromatic ring [62]. Direct attack on the aromatic ring results in the formation of styrene cis-glycol, followed by 3-vinylcatechol. Subsequently, 3-vinylcatechol undergoes ring-cleavage, yielding a muconic semialdehyde, which is further metabolized into pyruvate and acetaldehyde [63]. Calarnou et al. established that under abiotic conditions, photo- or thermo-oxidation processes generate multiple compounds, primarily short, oxidized chains resulting from main chain scissions, containing high levels of unsaturation and carbonylated functions. These short chains can be released into the water phase, potentially serving as substrates for microorganisms or as new cross-linking agents within the polymer matrix [64]. In a study by Olejnik et al., it was demonstrated that within a single cycle of bacterial culture, an average reduction of over 4% in rubber waste mass was observed [65]. Biodegradation investigations of natural rubber revealed a decrease in the number of cis-1,4 double bonds, the emergence of ketone and aldehyde groups, and the formation of two distinct bonding environments [66]. The overall degradation process was oxidative in nature, resembling chemical aging. The broader exothermal peaks observed in the DSC analysis of all samples subjected to 180 days of composting further support the notion of structural changes and increased cross-linking within the polymer matrix post-biodegradation. These broader peaks indicate a more complex thermal behavior, likely due to the presence of a heterogeneous polymer structure with varied degrees of cross-linking and degradation. 

Furthermore, the enhanced endothermal effects recorded for composted composites with higher biomass content may correlate with a higher retention of water, or decomposition by-products within the composite. The results are consistent with the SEM analysis that shows a higher porosity of the degraded samples with a higher content of biomass and IR analysis, which shows that adding biomass to the polymer matrix leads to higher hydrophilicity due to the formation of peptide bonds in the matrix. 

The possible explanation for these changes is that the polymer composite with microalgae biomass added forms cross-linked connections between the polymer chains and the polypeptide chain resulting from protein degradation, most probably through peptide bonds with the benzene units of the styrene blocks from the polymer matrix.

The observed changes in thermal behavior and the influence of biomass content have implications for the design and optimization of biodegradable materials. Considering the complex interactions between polymer matrices, biomass fillers, and biodegradation processes is essential for developing sustainable materials with tailored properties.

## 5. Conclusions

Algal biomass, such as that of *Chlorella vulgaris*, holds promise as a sustainable filler material for thermoplastic composites like poly(styrene-butadiene-styrene) (SBS) copolymers. Microalgae can be grown rapidly and sustainably, making it a renewable resource compared to traditional filler materials derived from fossil fuels, and algae-based fillers have the potential to help reduce the carbon footprint of composite materials, contributing to efforts to achieve net-zero carbon emissions for the thermoplastic polymer industry. By evaluating the behavior of loaded SBS composites with *Chlorella vulgaris* biomass on the industrial composting test, useful data about the feasibility of utilizing algae as a filler material in thermoplastic composites are obtained. The physical-chemical testing showed that the specific gravity of the samples decreased after the industrial composting test ended. Furthermore, there was a noticeable transformation of the material when the MFI was measured. The significant decrease in MFI post-biodegradation could limit the recyclability or reusability of these materials through conventional thermoplastic processing techniques. The observed changes confirm that the incorporation of *Chlorella* biomass not only affects the bio-degradation rate but also significantly impacts the physical properties of the composite material during degradation. Furthermore, the results obtained by the FTIR, SEM, and DSC analysis showed that the properties of composites that undergo the biodegradation process were influenced by the content of the microalgae biomass added to the composite. The physical-chemical changes observed, as well as FTIR, SEM, and DSC data, suggest that the biodegraded polymer structure exhibits increased cross-linking and higher porosity in samples with higher biomass content incorporated into the polymer matrix. A possible explanation of this behavior is that the polymer composites containing microalgae biomass form cross-linked connections between polymer chains and polypeptide chains resulting from protein degradation, adding more connections between polystyrene units of the polymer facilitated by peptide bonds with the benzene units of the styrene blocks within the polymer matrix.

This analysis of the physical and chemical changes in polymer composites induced by the addition of microalgae biomass and subsequent biodegradation highlights the complexity of designing sustainable and environmentally friendly thermoplastics based on SBS.

## Data Availability

The data presented in this study are available within the present article.
